# Antimicrobial activity of HL-60 cells compared to primary blood-derived neutrophils against *Staphylococcus aureus*

**DOI:** 10.1186/s12952-017-0067-2

**Published:** 2017-02-19

**Authors:** Ragheda Yaseen, Stefanie Blodkamp, Petra Lüthje, Friederike Reuner, Lena Völlger, Hassan Y. Naim, Maren von Köckritz-Blickwede

**Affiliations:** 1 Department of Physiological Chemistry, University of Veterinary Medicine Hannover, Buenteweg 17, 30599 Hannover, Germany; 2 Division of Clinical Microbiology, Karolinska Institutet, Karolinska University Hospital, Huddinge, Stockholm Sweden; 3 Research Center for Emerging Infections and Zoonoses (RIZ), University of Veterinary Medicine Hannover, Buenteweg 17, 30599 Hannover, Germany

**Keywords:** Neutrophil extracellular traps, Staphylococcus aureus, Myeloperoxidase, Phagocytosis

## Abstract

**Background:**

The human leukemia cell line HL-60 is considered an alternative cell culture model to study neutrophil differentiation and migration. The aim of this study was to characterize the suitability of HL-60 cells differentiated to neutrophil-like cells (nHL-60) as substitute for blood-derived human neutrophils to investigate the interaction of neutrophils with *Staphylococcus aureus*.

**Methods:**

For this purpose, antimicrobial activity, bacterial uptake, production of reactive oxygen species and the release of neutrophil extracellular traps (NETs) by nHL-60 cells were analyzed and compared to primary blood-derived neutrophils using *Staphylococcus aureus* as important human and animal pathogen.

**Results:**

Overall, the antimicrobial activities of nHL-60 cells were distinctly lower compared to blood-derived neutrophils. Furthermore, production of reactive oxygen species as well as NET formation was clearly impaired in nHL-60 cells.

**Conclusion:**

This study indicates that HL-60 cells are of limited usage as an alternative model to study antimicrobial functions of neutrophils against *Staphylococcus aureus*.

**Electronic supplementary material:**

The online version of this article (doi:10.1186/s12952-017-0067-2) contains supplementary material, which is available to authorized users.

## Introduction

The usage of primary blood-derived neutrophils to study host-pathogen interactions in vitro has important limitations to the experimental design: One limitation is the total number of cells that can be harvested from fresh blood. Furthermore, differences between individual donors may influence comparison between experiments. In addition, the isolation of neutrophils from whole blood requires specific equipment and is laborious, since primary neutrophils are short-living cells and may undergo apoptosis rapidly. A cell line-based model to substitute blood-derived neutrophils is therefore highly wanted. The human leukemia cell line HL-60 is considered an alternative cell culture model to study neutrophil functions. In this case, DMSO and all trans-retinoic acid (RA) are widely used to differentiate HL-60 cells to neutrophil-like cells [1, 2]. Although the differentiated neutrophil-like cells show many characteristics of primary neutrophils, the differentiation is somewhat incomplete and defective [3–5].

Neutrophils possess different antimicrobial activities to fight against invading pathogens. The most prominent one is phagocytosis, where pathogens are internalized and killed intracellularly by non-oxidative and oxidative mechanisms [6]. Another strategy is degranulation, the release of the granular content, e.g. antimicrobial peptides, into the extracellular space. More recently, the formation of extracellular traps (ETs) by neutrophils [7] and other leukocytes [8] has been discovered as an additional mechanism to entrap and kill pathogens extracellularly. Key mediators to trigger phagocytosis as well as formation of neutrophil ETs (NETs) are reactive oxygen species (ROS), generated by the membrane-bound NADPH oxidase enzyme complex.

The goal of this study was to characterize the antimicrobial activity of differently differentiated HL-60 cells against the pathogen *Staphylococcus aureus* in comparison to primary human blood-derived neutrophils, with special emphasis on the formation of NETs. *S. aureus* is one of the leading cause of serious bacterial infections in the United States and many other developed countries. The bacterium has the ability to produce abscesses in every tissue and organ system that is colonized. Nowadays, severe increases in diseases caused by methicillin-resistant *S. aureus* (MRSA) in human as well as animals have occurred. The fact MRSA plays an important role in health care and community setting leads to an ominous threat to public health [9, 10].

## Methods

### Bacterial strains and growth conditions

For testing antimicrobial activity of cells, *S. aureus* Newman was used; for NET induction assays, a nuclease-deficient derivative of *S. aureus* USA 300 LAC (*S. aureus* AH1787) was used [11]. The absence of bacterial nuclease activity ensured to capture total NET formation without interference with NET degradation. Bacteria were grown in brain heart infusion (BHI) medium at 37 °C shaking. An overnight culture was diluted 1:100 into fresh medium and grown to mid-logarithmic phase (OD_600_ = 0.5). Bacteria were then harvested by centrifugation, suspended in PBS and adjusted to the desired concentration by optical density at 600 nm. Further dilutions were prepared in cell culture medium.

### Cultivation and differentiation of HL-60 cells

The myeloid leukemia cell line HL-60 was propagated in RPMI 1640 medium, supplemented with 10% fetal bovine serum (FBS) heat-inactivated at 56 °C and 1% penicillin/streptomycin (all from PAA). To induce a neutrophil-like phenotype, cells were treated with either 1.25% DMSO for 3 days [12], 1.25% DMSO for 4 days [13] or 1 μM RA for 4 days [5] without medium change, reaching a maximum cell count of 1 × 10^6^ cells/ml. For experiments, differentiated cells were collected by centrifugation for 10 minutes at 118 × *g*, washed once with PBS and finally adjusted to a density of 2 × 10^6^ cells/ml in RPMI 1640 supplemented with 2% nuclease-free FBS (heat-inactivated at 70 °C). HL-60 cells cultured and differentiated according to this protocol will further be referred to as nHL-60.

### Isolation of human blood-derived neutrophils

Human neutrophils were isolated from freshly taken venous blood from healthy donors in agreement with the local ethical board (Medizinische Hochschule Hannover, ethical agreement 3295–2016) by density gradient centrifugation using PolymorphPrep according to the manufacturer’s protocol (Axis-Shield). Neutrophils were adjusted to a density of 2 × 10^6^ cells/ml in RPMI 1640 supplemented with 2% nuclease-free FBS.

### Control assay to determine dead cells

Differentiated nHL-60 cells or primary neutrophils were incubated for 4 h at 37 °C, and 5% CO_2_. Samples were stained with 0.4 mg/ml trypan blue as indicator for dead cells; based on their positive staining the percentage of dead cells was calculated compared to total cell count using light microscopy. These control experiments revealed that within the 4 hours of experiments here, less than 5% cells were found to be dead by tryphan exclusion assay for all types of cells.

### Antimicrobial activity assay

To determine the antibacterial activity of nHL-60 cells or neutrophils, cells were co-incubated with bacteria at a multiplicity of infection (MOI) of 2 in a final volume of 500 μl in 48-well non-treated cell culture plates. All incubations were carried out at 37 °C and 5% CO_2_ in a humidified incubator. Prior to infection, cells were pre-stimulated for 20 minutes with phorbol 12-myristate 13-acetate (PMA; 25 nM). Control cells received the vehicle (DMSO) in the same dilution. Bacteria were then added to the cells, the plates were centrifuged for 5 minutes at 472 × *g* and incubated for 30 minutes. Cells were lysed by addition of 50 μl of 0.25% Triton X-100 in PBS and serial dilutions were plated on Todd-Hewitt agar plates for viable count. All conditions were analyzed in duplicate. Results were expressed as surviving bacteria compared to bacterial growth under the same conditions in the absence of cells.

### Determination of bacterial uptake

BioParticles®-Tetramethylrhodamine conjugate from *S. aureus* Wood strain (Sigma) was co-incubated with neutrophils at a MOI of 30 for 30 min at 37 °C in 5% CO2. Afterwards, the cells were washed with PBS to remove unbound bacteria and filtered through a Sysmex CellTrics® 30 µm filter. FITC fluorescence as a marker for phagocytosis was measured using an Attune NxT Flow Cytometer (Thermo Fisher Scientific). The percentage of cells that were positive for bacterial uptake compared to respective negative control was determined. Furthermore, mean red fluorescence intensity per neutrophil (Gx-Mean of BL-2) was recorded and represents the mean relative phagocytosis of FITC-labeled *S. aureus* per neutrophil.

### Formation of ROS

ROS was determined by change in fluorescence resulting from oxidation of the fluorescent probe DCF. Briefly, 5 × 10^5^ cells/250 μL were treated with PMA or vehicle control DMSO for 0.5 hours at 37 °C in 5% CO_2_. After incubation, cells were then incubated with fluorescent dye DCF (10 μM) for 10 min at room temperature. The relative ROS formation was analyzed using the fluorescence detector FL-1 of an Attune NxT Flow Cytometer. Mean green fluorescence intensity of all (x-Mean of BL-1) was recorded and represents the mean ROS production.

### NET induction assay

The capacity of nHL-60 cells and blood-derived neutrophils to form NETs was assessed after stimulation with PMA and *S. aureus* AH1787. Cells were seeded on 8-mm cover slips coated with poly-L-lysin, stimulated with 25 nM PMA and/or bacteria at a MOI of 2 as indicated and centrifuged for 5 minutes at 472 × *g*. The plates were then incubated at 37 °C and 5% CO_2_ in a humidified incubator for 1, 2, 3 or 4 hours. Cells were fixed by addition of paraformaldehyde (PFA) in PBS to a final concentration of 4% PFA. For all conditions, preparations were performed in duplicate.

### NET visualization and quantification

Fixed cells were washed three times with PBS and permeabilized and blocked with 2% BSA in 0.2% Triton X-100/PBS for 45 minutes at room temperature. Incubation with a mouse monoclonal anti-H2A-H2B-DNA complex (clone PL2-6 [14], 0.5 μg/ml in 2% BSA in 0.2% Triton X-100/PBS) was carried out overnight at 4 °C, followed by washing (3 times with PBS) and subsequent incubation with an AlexaFluor488-labelled anti-mouse antibody for 45 minutes at room temperature. After washing, slides were mounted in ProlongGold antifade including DAPI and analyzed by confocal fluorescence microscope using a Leica DMI6000CS confocal microscope with a HCXPLAPO 40 × 0.75–1.25 oil objective. Preparations with an isotype control antibody were used for setting adjustment. For each preparation, three randomly selected images were acquired and used for quantification of NET producing cells. Data were expressed as percentages of NET-forming. The mean value derived from *n* = 6 images for each condition per experiment was used for statistical analysis.

### Statistical analysis

The average values derived from independent experiments performed in duplicate were used for statistical analysis and are depicted as mean and standard error of the mean (SEM). Since pooling of control data revealed normal distribution of data values by Kolmogorov Smirnov test as expected for in vitro experiments, parametric tests were used for statistical analysis: In general, comparisons between stimulated and non-stimulated cells of equally differentiated cells were performed by using unpaired, one-tailed *t*-test, if not indicated otherwise. Comparisons between differently differentiated cells or different cell types were performed by using unpaired, two-tailed *t*-test, if not indicated otherwise. In both cases, differences with *P* < 0.05 were considered statistically significant.

## Results

### nHL-60 cells exhibit low antimicrobial activity

**Fig. 1:**
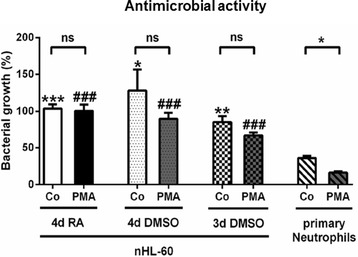
Antimicrobial activity of nHL-60 cells and blood-derived neutrophils against *S. aureus* Newman. The figure shows the antimicrobial activity of nHL-60 cells and blood-derived neutrophils against *S. aureus* Newman with and without PMA-stimulation. Results from three independent experiments are depicted as mean and SEM (*n* = 3). Comparisons between unstimulated (Co) and PMA-stimulated cells (PMA) were performed by paired, one-tailed *t*-test; *P < 0.05, and comparisons between nHL-60 cells and blood-derived neutrophils were performed by unpaired, two-tailed t-test; **P < 0.01, ***P < 0.001 for the comparisons of the controls with control of primary neutrophils and ### *P* < 0.001 for comparison of PMA stimulation with respective PMA-stimulated primary neutrophils

To investigate whether differentiated HL-60 (nHL-60) cells act antibacterial, we co-incubated *S. aureus* Newman with nHL-60 (Fig. 1). Prior to infection, cells were stimulated with PMA, a widely used neutrophil activator, or left untreated. After differentiation with DMSO for 3 days, bacterial growth was slightly reduced (85.3%) compared to incubation of bacteria in cell-free medium (100%) and the antibacterial activity could be enhanced by PMA-stimulation (66.8%), although this effect did not reach significance. Cells differentiated with RA or DMSO for 4 days were completely not antimicrobial active against *S. aureus*, even if pre-stimulated with PMA. In contrast, primary blood-derived neutrophils showed significantly increased antimicrobial activity against *S. aureus* and exhibited a reduction of bacterial growth to 36.1% in absence of PMA and 16.3% in the presence of PMA (Fig. 1). Thus, in general, nHL-60 cells did exhibit significantly less antimicrobial activity against *S. aureus* compared to human blood-derived neutrophils, regardless of the differentiation method used for nHL-60 cells. The best effect, even though not reaching statistical significance, was achieved by differentiation with DMSO for 3 days. Therefore, this condition was used for all following experiments.

### nHL-60 cells show impaired formation of ROS

**Fig. 2:**
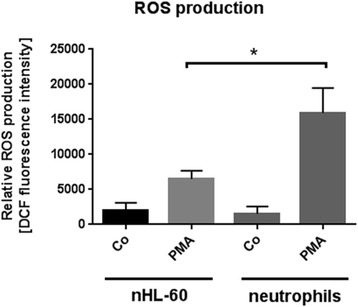
ROS-production by nHL-60 cells and blood-derived neutrophils. This figure shows the relative production of ROS by 3 day DMSO-differentiated nHL-60 cells and primary neutrophils in the presence and absence of PMA. Results from three independent experiments are depicted as mean and SEM (*n* = 3). Comparison between primary neutrophils and nHL-60 cells was performed by unpaired, two-tailed *t*-test; *P < 0.05

Since stimulation with PMA triggers the formation of reactive oxygen species by NADPH-oxidase, we next quantified the formation of ROS using a ROS-sensitive fluorescent dye 2′, 7′-dichlorofluorescein (DCF). In good correlation to the distinctly lower antimicrobial activity of nHL-60 cells, also the formation of ROS was significantly reduced in nHL-60 cells compared to blood-derived neutrophils (Fig. 2).

### No difference in bacterial uptake comparing nHL-60 cells with neutrophils

**Fig. 3:**
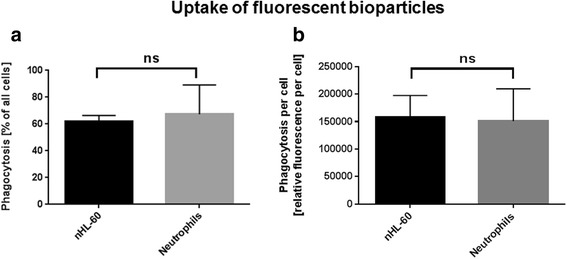
Uptake of fluorescent *S. aureus* bioparticles by nHL-60 cells and blood-derived neutrophils. This figure shows the uptake of fluorescently labeled *S. aureus* bioparticles by 3 day DMSO-differentiated nHL-60 cells and primary neutrophils, indicated as percentage of phagocytosis (**a**) or relative phagocytosis per cell (**b**). Results from three independent experiments are depicted as mean and SEM (*n* = 3). Comparison between primary neutrophils and nHL-60 cells was performed by unpaired, two-tailed *t*-test; *P < 0.05; ns = not significant

The formation of ROS is involved in intracellular killing after bacterial uptake by phagocytosis as well as extracellular killing by NETs. Therefore, as a next step we investigated the bacterial uptake of fluorescent bioparticels as marker for phagocytosis by quantitative flow cytometry: As shown in Fig. 3, there is no difference in bacterial uptake, neither when quantifying the percentage of cells that are positive for fluorescent bioparticles (Fig. 3a), nor on a single cell basis (Fig. 3b).

### nHL-60 cells form less NETs compared to neutrophils

**Fig. 4:**
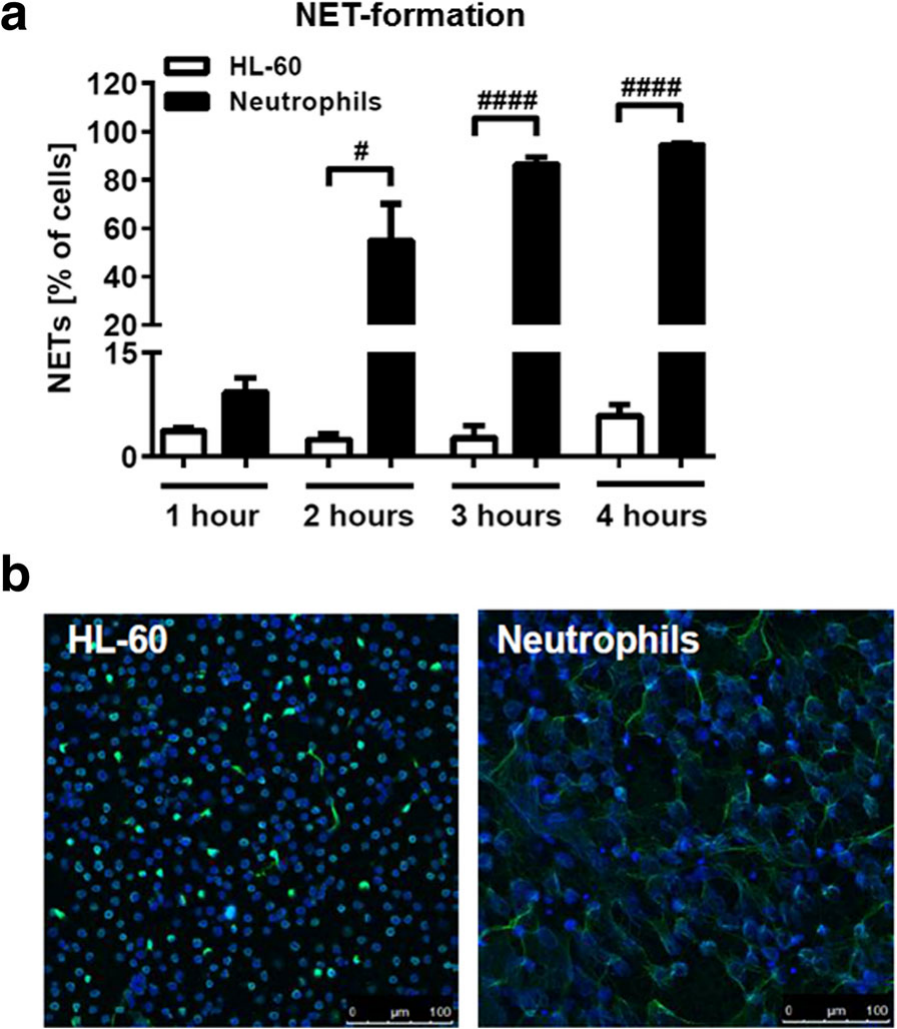
NET-formation of PMA-stimulated primary neutrophils and 3 days DMSO-differentiated HL-60 cells. **a** NET formation by HL-60 cells differentiated with DMSO for 3 days and blood-derived neutrophils was assessed after stimulation with PMA for indicated periods of time. Results from 3 to 4 experiments are depicted as mean and SEM (*n* = 3–4). Comparison between nHL-60 cells and neutrophils at each time point was performed by unpaired, two-tailed *t*-test; #*P* < 0.05, ####*P* < 0.0001. **b** Representative images from experiments shown in (**a**). nHL-60 or blood-derived neutrophils were stimulated with PMA for 4 hours, fixed and stained with an antibody directed against histone-DNA-complexes and a secondary AlexaFluor488-labelled anti-mouse antibody (*green*). The nuclei were stained with DAPI (*blue*). The scale bar is 100 μm

**Fig. 5:**
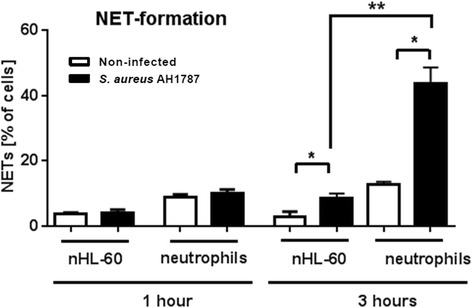
NET-formation of nHL-60 cells compared to primary neutrophils infected with *S. aureus.* nHL-60 cells (3 days DMSO) were infected with the nuclease deficient *S. aureus* AH1787 strain and compared to infected primary blood-derived neutrophils (Neutrophils). Results from three experiments are shown as mean and SEM. Comparisons between non-infected and infected cells were performed by paired, one-tailed *t*-test; *P < 0.05, and comparisons between nHL-60 cells and blood-derived neutrophils were performed by unpaired, two-tailed t-test; **P < 0.01

Next we investigated the ability of nHL-60 cells to form NETs in response to chemical (PMA) or biological (*S. aureus*) stimuli. While chemical PMA-induced NET formation in blood-derived neutrophils was already evident after 2 hours and complete (with more than 95% of the cells) after 4 hours of incubation, only single and significantly less nHL-60 cells released NETs at similar time points (Fig. 4). Similarly, also the biological stimulation with the nuclease-deficient model bacterium *S. aureus* AH1787 revealed significantly less NET release by nHL-60 cells compared to blood-derived neutrophils (Fig. 5). A maximum of 28% of NET-release was reached after 4 h of co-incubation nHL-60 cells with PMA and *S. aureus* AH1787 (Additional file 1: Figure S1). Differentiation with RA did also not substantially increase the ability to form NETs (Additional file 2: Figure S2). Actually, after differentiation with RA the amount of produced NETs was decreased (11.8% and 7.5% after stimulation with PMA only and additional *S. aureus* infection, respectively). A longer differentiation with DMSO on the other hand leads to a statistically significant increase in NET-production after 4 hours of incubation. Nevertheless, with only 25% NETs it is still to a much lower extent compared to blood-derived neutrophils which produce almost 100% NETs after 4 hours of stimulation.

## Discussion

In this study we aimed to examine whether differentiated HL-60 cells could provide a model for replacing primary blood-derived neutrophils for in vitro experiments to investigate antimicrobial functions. Compared to neutrophils, the overall antibacterial activity of nHL-60 cells against the model organism *S. aureus* was greatly reduced, even in the presence of the chemical stimulant PMA. Based on the results from this study, we conclude that the major antibacterial mechanisms exhibited by neutrophils, i.e. intracellular lysosomal killing and NET formation, are diminished in nHL-60 cells.

It is an established fact that HL-60 cells do not harbor the entire arsenal of granules, making their differentiation somewhat incomplete. As a marker for cell differentiation myeloperoxidase expression of the differentiated HL-60 cells was assessed in this study (Additional file 3: Figure S3). The data demonstrated almost 100% of myeloperoxidase-positive nHL-60 cells, indicating a neutrophil-like phenotype. Nonetheless, the overall antimicrobial functions were decreased in nHL-60. Since HL-60 cells lack secondary granules and secretory vesicles, which contain the vast proportion of *b*_558_, the membrane-bound component of the NADPH oxidase enzyme complex, their ability to produce ROS might be impaired. Supportive for this hypothesis is a study conducted by Nordenfelt et al., 2009 [5]. Using *Streptococcus pyogenes* as a model organism, the authors conclude that HL-60 cells can replace neutrophils in models which do not rely on respiratory burst activity, pointing towards reduced capacity of HL-60 cells to generate ROS. We confirmed this phenomenon by measuring formation of ROS in response to PMA (Fig. 5). The presence of myeloperoxidase would not overcome this issue, as it acts downstream of NADPH. However, reports regarding the presence of NADPH oxidase and ROS generation in HL-60 cells are controversial [15]. Besides *b*_558_ the antimicrobial peptide cathelicidin hCAP18/LL-37 is stored in secondary granules. Hence, an absence of these granules implicates also the lack of LL-37 in HL-60 cells. Furthermore, An et al. (2005) showed that peripheral blood cells from patients with acute myeloid leukemia do not produce this cathelicidin, even though gene-specific mRNA is detectable [3]. In HL-60 cells, this deficiency could not be converted by differentiation using RA [3]. Primary granules, carrying most of the antimicrobial peptides including neutrophil defensins, on the other hand can be found in HL-60 cells [4]. Nevertheless, the lacking of LL-37 and impaired ROS production might be explanations for the reduced killing of *S. aureus* by nHL-60 cells in comparison to primary neutrophils.

Consistent with the results of other studies [15–17], also in these experiments nHL-60 cells produced NETs, although to much lower extent than primary neutrophils. A putative defect in the neutrophil NADPH oxidase enzyme complex appears also a feasible reason for the failure of nHL-60 cells to efficiently produce NETs. It is known that ROS production by NADPH oxidase is essential for an efficient NET production, [18] therefore a lack of this enzyme would lead to a downgraded NET formation. A partial NET-formation in response to *S. aureus* may be explained by the fact, that an additional ROS-independent mechanism of NET-formation has been described [19]. In addition, neutrophil elastase, a component in the primary granules, has recently been identified being indispensible for NET formation [20]: Lysates of HL-60 cells failed to induce NET release from isolated nuclei, indirectly indicating that the primary granules of HL-60 cells lack this enzyme.

## Conclusion

These findings together show that the development of neutrophil characteristics is insufficient in HL-60 cells: HL-60 cells after chemical differentiation with DMSO or RA do not exert similar antibacterial activities compared to blood-derived neutrophils. Thus we conclude that, HL-60 cells differentiated with DMSO or RA are of limited value to replace primary cells in in vitro experiments to investigate host-pathogen interactions, especially in case of *S. aureus*.

## Additional files

Additional file 1: Figure S1. NET-formation of PMA-stimulated nHL-60 cells infected with a *S. aureus* USA 300 LAC strain. (A) PMA-stimulated nHL-60 cells (3 days DMSO) were infected with the nuclease deficient *S. aureus* AH1787 strain and compared to non-infected PMA-stimulated cells. Results from three experiments are shown as mean and SEM. Comparison between non-infected and infected cells was performed by unpaired one-tailed *t*-test; **P* < 0.05. (B) Representative images from experiments shown in (A). PMA-stimulated nHL-60 without (non-infected) or infected with *S. aureus* AH1787 for up to 4 hours were fixed and stained with an antibody directed against histone-DNA-complexes and a secondary AlexaFluor488-labelled anti-mouse antibody (green). The nuclei were stained with DAPI (blue). The scale bar is 100 μm. (TIF 248 kb)
Additional file 2: Figure S2. NET-formation by differently differentiated HL-60 cells after 4 hours. NET formation by differently differentiated HL-60 cells with and without PMA stimulation and *S. aureus* infection after 4 hours. The results of three independent experiments are shown as mean and SEM. Comparisons between none-stimulated, PMA-stimulated and *S. aureus*-infected cells and between differently differentiated HL-60 cells were performed by unpaired, one-tailed *t*-test; **P* < 0.05. (TIF 72 kb)
Additional file 3: Figure S3. Percentage of myeloperoxidase-positive cells of differently differentiated HL-60 cells. (A) Percentage of myeloperoxidase-positive cells of HL-60 cells after 2 hours of incubation with PMA. The results of three independent experiments are shown as mean and SEM. Comparisons between the differently differentiated HL-60 cells were performed by unpaired, one-tailed *t*-test. (C-D) Representative fluorescence micrographs of data shown in (A). nHL-60 cells stained with an antibody directed against myeloperoxidase and a secondary AlexaFluor488-labelled anti-rabbit antibody (green) (C). The nuclei were stained with DAPI (blue) (B) and an overlay is shown in (D). (TIF 413 kb)

## Abbreviations

RA: All trans-retinoic acid
BHI: Brain heart infusion
FBS: Fetal bovine serum
MOI: Multiplicity of infection
NETs: Neutrophil extracellular traps
PFA: Paraformaldehyde
ROS: Reactive oxygen species
SEM: Standards error of the mean
