# Supplementary figures and images for: Antimicrobial activity of HL-60 cells compared to primary blood-derived neutrophils against Staphylococcus aureus
Source: J Negat Results Biomed. 2017 Feb 19;16:2. doi: 10.1186/s12952-017-0067-2 (PMC5316427; doi:10.1186/s12952-017-0067-2)

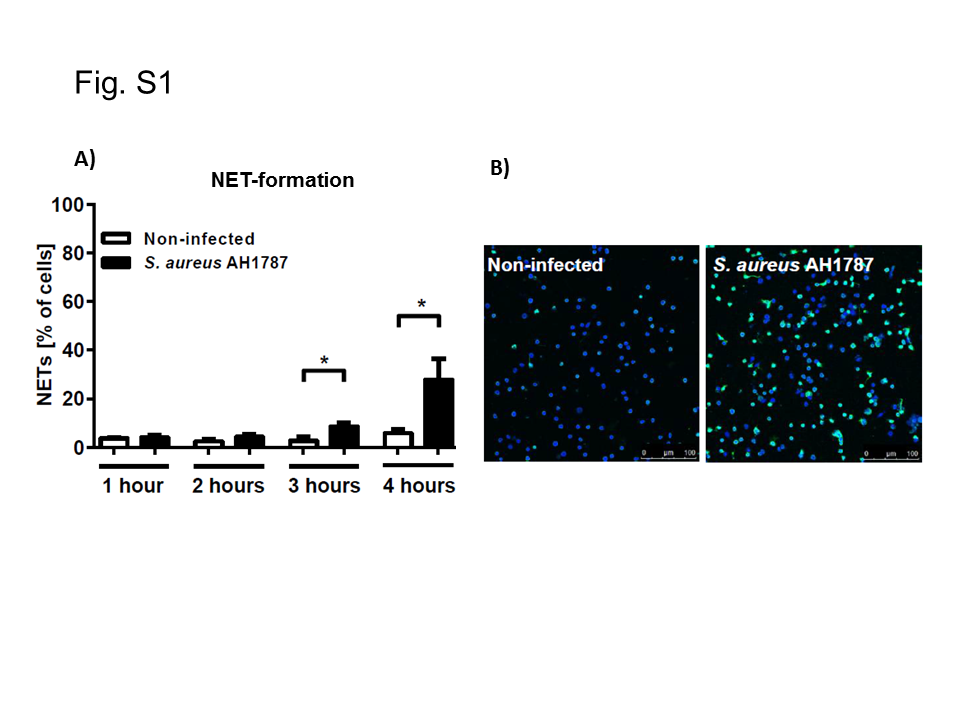

Supplement: Additional file 1: Figure S1. — NET-formation of PMA-stimulated nHL-60 cells infected with a S. aureus USA 300 LAC strain. (A) PMA-stimulated nHL-60 cells (3 days DMSO) were infected with the nuclease deficient S. aureus AH1787 strain and compared to non-infected PMA-stimulated cells. Results from three experiments are shown as mean and SEM. Comparison between non-infected and infected cells was performed by unpaired one-tailed t-test; *P < 0.05. (B) Representative images from experiments shown in (A). PMA-stimulated nHL-60 without (non-infected) or infected with S. aureus AH1787 for up to 4 hours were fixed and stained with an antibody directed against histone-DNA-complexes and a secondary AlexaFluor488-labelled anti-mouse antibody (green). The nuclei were stained with DAPI (blue). The scale bar is 100 μm. (TIF 248 kb) [file 12952_2017_67_MOESM1_ESM.tif]

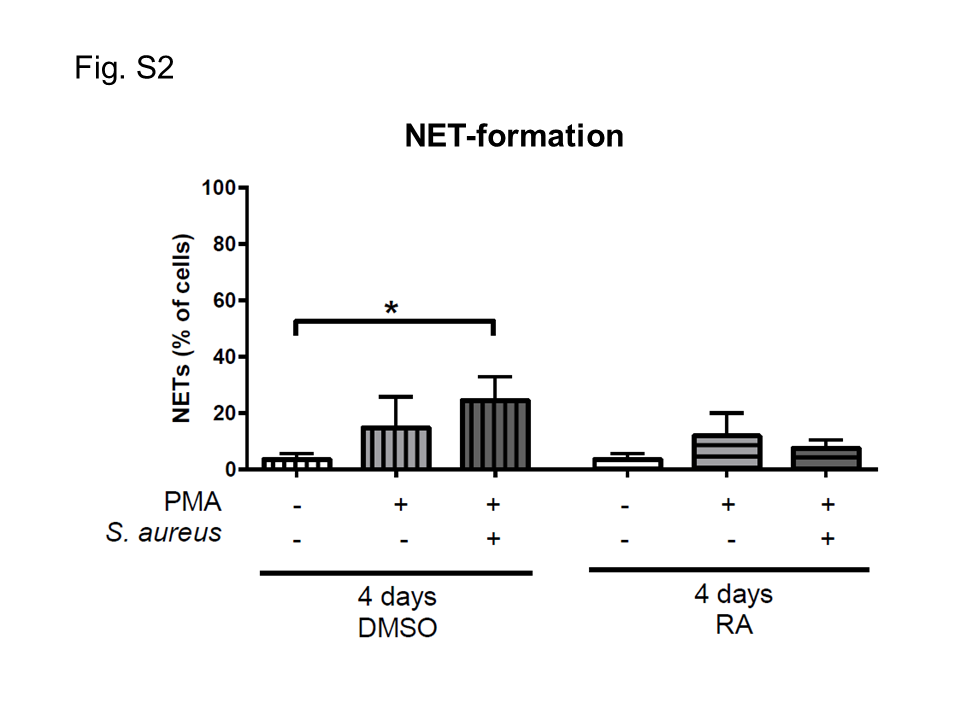

Supplement: Additional file 2: Figure S2. — NET-formation by differently differentiated HL-60 cells after 4 hours. NET formation by differently differentiated HL-60 cells with and without PMA stimulation and S. aureus infection after 4 hours. The results of three independent experiments are shown as mean and SEM. Comparisons between none-stimulated, PMA-stimulated and S. aureus-infected cells and between differently differentiated HL-60 cells were performed by unpaired, one-tailed t-test; *P < 0.05. (TIF 72 kb) [file 12952_2017_67_MOESM2_ESM.tif]

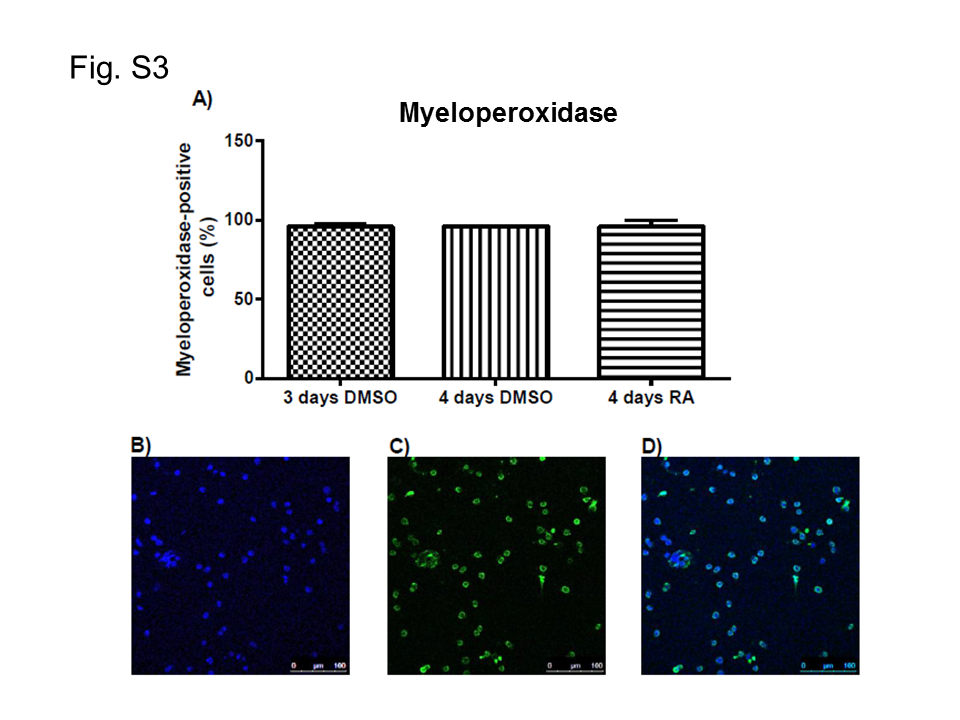

Supplement: Additional file 3: Figure S3. — Percentage of myeloperoxidase-positive cells of differently differentiated HL-60 cells. (A) Percentage of myeloperoxidase-positive cells of HL-60 cells after 2 hours of incubation with PMA. The results of three independent experiments are shown as mean and SEM. Comparisons between the differently differentiated HL-60 cells were performed by unpaired, one-tailed t-test. (C-D) Representative fluorescence micrographs of data shown in (A). nHL-60 cells stained with an antibody directed against myeloperoxidase and a secondary AlexaFluor488-labelled anti-rabbit antibody (green) (C). The nuclei were stained with DAPI (blue) (B) and an overlay is shown in (D). (TIF 413 kb) [file 12952_2017_67_MOESM3_ESM.tif]
